# What Drives Farmers to Make Top-Down or Bottom-Up Adaptation to Climate Change and Fluctuations? A Comparative Study on 3 Cases of Apple Farming in Japan and South Africa

**DOI:** 10.1371/journal.pone.0120563

**Published:** 2015-03-30

**Authors:** Mariko Fujisawa, Kazuhiko Kobayashi, Peter Johnston, Mark New

**Affiliations:** 1 The University of Tokyo, Yayoi 1-1-1, Bunkyo-ku, Tokyo, 113–8657, Japan; 2 African Climate and Development Initiative (ACDI), University of Cape Town, Private Bag x3, Rondebosch 7701, Cape Town, South Africa; 3 Climate System Analysis Group (CSAG), University of Cape Town, Private Bag x3, Rondebosch 7701, Cape Town, South Africa; University of Vermont, UNITED STATES

## Abstract

Agriculture is one of the most vulnerable sectors to climate change. Farmers have been exposed to multiple stressors including climate change, and they have managed to adapt to those risks. The adaptation actions undertaken by farmers and their decision making are, however, only poorly understood. By studying adaptation practices undertaken by apple farmers in three regions: Nagano and Kazuno in Japan and Elgin in South Africa, we categorize the adaptation actions into two types: farmer initiated bottom-up adaptation and institution led top-down adaptation. We found that the driver which differentiates the type of adaptation likely adopted was strongly related to the farmers’ characteristics, particularly their dependence on the institutions, e.g. the farmers’ cooperative, in selling their products. The farmers who rely on the farmers’ cooperative for their sales are likely to adopt the institution-led adaptation, whereas the farmers who have established their own sales channels tend to start innovative actions by bottom-up. We further argue that even though the two types have contrasting features, the combinations of the both types of adaptations could lead to more successful adaptation particularly in agriculture. This study also emphasizes that more farm-level studies for various crops and regions are warranted to provide substantial feedbacks to adaptation policy.

## Introduction

Agriculture is one of the most vulnerable sectors to climate change [1], and responding to both extreme events and climatic variability is particularly challenging for farmers [2]. As the farmers’ responses to climate change are being analyzed (see review [1]], there emerges an optimism that successful adaptations: “actions of adjusting practices, processes, and capital in response to the actuality or threat of climate change [3]” will take place in the future [4].

In reality, however, few consistencies have been found among studies of adaptation practices by farmers to deal with climate change [5]. For example, crop diversification has been commonly recognized as a potential response to climatic variability and change, but, its adoption by farmers for this purpose is not well understood [5]. Farmers’ adaptation to climate change is modulated by their exposure to multiple and interacting stressors [6] such as changes in agricultural policy, labor conditions, cost of inputs and market prices, and their decision making is often largely affected by these various non-climatic stimuli. In addition, farmers’ individual adaptation goals often differ within and between regions, yet these goals are seldom stated explicitly [7], which makes the study of climate change adaptation in agriculture a scientific challenge. Furthermore, only a limited number of studies are based on actual field-based observations of farm-level adaptation responses [8], while many existing studies refer to the adaptation by farmers.

In order to reveal the drivers affecting farmers’ decision making with respect to climate change among multiple exposures, we studied the apple (*Malus pumila var*. *domestica*) farmers’ perceptions of, and adaptation to the risks in three apple production areas: Kazuno and Nagano in Japan, and Elgin in South Africa. The three regions differ from each other with respect to their climatic suitability for apple production: Kazuno is climatically well suited and will be so in 2060 as well [9]. Nagano is climatically suited, but negative effects of high temperature such as paler color and softening of fruits have recently been observed [10], and the climate would become too warm in 2060 [9]. In Elgin, the climate is already close to the warmer margin with insufficient winter chill units [11], and further warming would push this region beyond the critical threshold where apple production could no longer be commercially viable [12].

According to the climatic suitability, it may appear less likely that adaptive actions are taken in Kazuno than the other two regions. In fact, however, apple farmers in Kazuno made a drastic change by introducing peaches (*Prunus persica var*. *vulgaris*), a species better suited to warm climate than apple, and now Kazuno is recognized as one of the northernmost peach production areas in Japan [13]. In Elgin, on the other hand, the farmers maintain apple production by changing cultivars to those with lower chill unit requirements and higher tolerance against sunburn [14]. In Nagano, some farmers have adopted technical remedies against the poor coloring and have changed cultivars, like the farmers in Elgin, to minimize the negative effects of warming. Others managed to establish a niche for their apples without having to adjust the appearance quality of the fruits to the existing market (details in following sections). It is therefore evident that the farmers’ adaptations are not a simple function of the pressures from climate change or other stressors.

The drivers and parameters that eventually lead farmers to the various adaptive actions are of primary interest to us. To this end, we set three objectives for this study. The first is to identify the drivers that prompted and differentiated farmers’ perceptions and adaptations at the individual level. The second objective is to appraise the roles of institutions in the adaptation process particularly when collective actions are taken at a regional scale, and to see the linkage between the individual and regional adaptations [15]. The third objective is to categorize the adaptation actions according to its timing, intent [16] and occurrence, i.e. top-down or bottom-up. We thus try to get better understanding of decision making process of the farmers who actually perform the adaptations, and eventually to facilitate institutional support for agricultural adaptations with a greater chance of success. The analysis was conducted using the materials obtained in our own cases-studies in the three regions: Kazuno [13], Nagano [17] and Elgin [14]

This article has a further six sections. Section 2 outlines the three study regions and their climatic suitability for apple production. Section 3 describes the methodologies, and section 4 presents the results of the study on farmers’ perceptions of climatic and non-climatic stimuli and actions taken against the stimuli. Section 5 discusses the process of adaptation from two points of view: the farmers’ actions and the roles of institutional support. Section 6 concludes the article by giving implications for further studies.

## Apple Production in the Study Areas and the Climate Therein

Japan and South Africa have similar size of apple production: Japan produces 786 500 tonnes of apples annually and is ranked 16^th^ in world production, while South Africa produces 724 200 tonnes and is ranked 18^th^ in 2010 [18]. The two countries differ from each other in other aspects of the apple production, however. The main cultivars in Japan are Fuji (54%) and Tsugaru (11%) followed by Golden Delicious (8%) and Jonagold (7%) [19]. Almost all the apples are for domestic consumption with only 2.3% being exported in 2010 [19]. In South Africa, the main cultivars are Golden Delicious (25%), Granny Smith (21%), Royal Gala (15%), and Starking (14%) followed by Cripps’ Pink (9%) and Fuji (7%) [20]. 41.6% of the total apple production is for export, 30.2% is for local market and 29.7% for processing (local use and export) [20].

**Fig 1:**
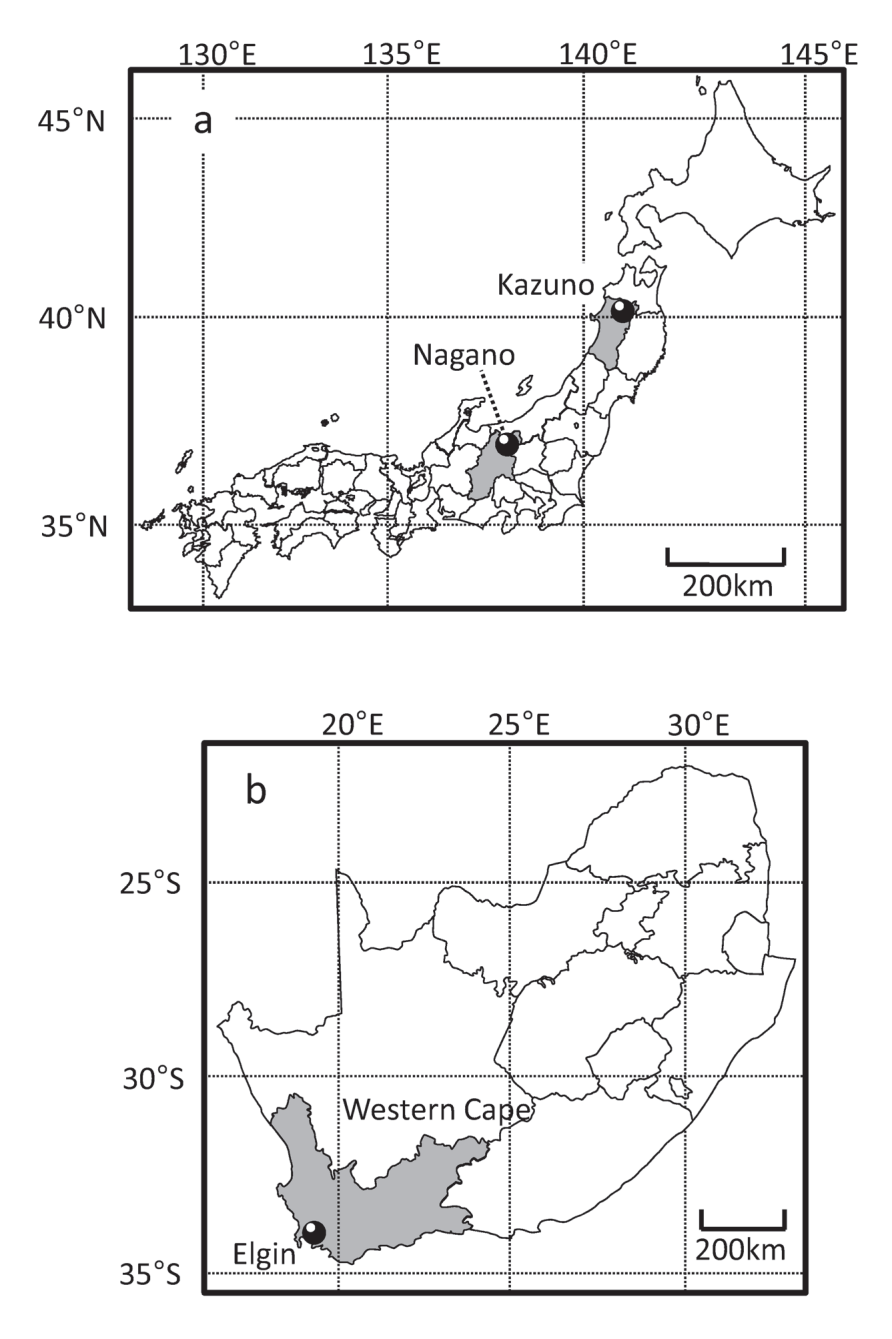
Three study regions: Kazuno and Nagano in Japan (a) and Elgin in Western Cape, South Africa (b) shown with dots.

The city of Kazuno is located in Akita prefecture, northern Japan (Table 1 and Fig 1-A). Apple production in Kazuno began around 1890, when American apple varieties were first brought to Japan. It has been one of the major apple production areas with quality fruits being highly valued at the market, owing to the suitable environmental conditions. The climate is categorized as a subarctic zone with a mean annual air temperature of 9.4 °C (averaged over the past 30 years from 1981 to 2010) and a warming trend of 0.26 °C/ decade (p<0.05) has been identified [21]. Most, if not all, farmers in this area belong to a farmers’ cooperative. There are about 350 apple growers in Kazuno and their average growing area of apple is 0.57 ha [22].

**Table 1:** The basic information of the study sites.

Study region	Country	Latitude/ longitude	Annual mean air	Number of farmers interviewed		
			temperature (°C)	Total	Co-op^a^	Non-co-op^a^
Kazuno	Japan	40° 13' N/ 140° 46' E	9.4	40	19	21
Nagano	Japan	36° 39' N/ 138° 19' E	12.1	26	13	13
Elgin	South Africa	34° 08' S/ 19° 02' E	16.3	11	11	0

Annual mean temperature is average across the last 30 years.

^a^ A farmer is referred to as a ‘Co-op farmer’ when more than a half of their products are sold through the farmers’ cooperative or an equivalent institution, otherwise the farmer is a ‘Non-co-op farmer’.

Nagano Prefecture is the second largest apple producer in Japan with its harvest contributing to 18% of the national harvest as of 2010 [19]. For this study, we chose a region consisting of two townships of Suzaka and Obuse, and a village of Takayama in the north of the prefecture (Fig 1-A). The farmers in this region belong to a common farmers’ cooperative, through which the members can ship their products to the market. They began apple cultivation around 1950 and have made apple as the dominant crop. It is one of the southernmost production areas in Japan, with a mean annual air temperature 11.9 °C (averaged over the past 30 years from 1981 to 2010) and a warming trend of 0.39 °C/decade (p<0.01) [21]. This study region has about 1200 apple growers, whose average apple growing area is 0.36 ha [22].

Elgin is located in Western Cape and one of the largest apple production areas in South Africa (Table 1 and Fig 1-B). In 2011, about 90% of apples in South Africa were produced in Western Cape, and about 43% of the national production was in Elgin [20]. The first commercial deciduous fruit orchards were established in the Elgin valley around 1900 and these orchards were the precursor to the development of the apple industry, which remains the economic backbone in the area. There are about 130 apple farmers in the region, and they also produce pears, nectarines, peaches and grapes. The average individual farm area planted to apple is estimated to be around 34 ha [23]. For the period from 1973 to 2009, the mean annual air temperature was 16.3 °C, and a warming trend of 0.55 °C/ decade (p<0.001) has been found in the annual temperature in Elgin [24].

The climate in the study regions differs clearly from each other with respect to the suitability for apple production as defined in Japan [25] viz.,

Annual mean air temperature to be between 7 °C and 13 °C, and

Temperature being below 7.2 °C for more than 1400 hours for dormancy release.

The annual mean temperature in Kazuno (9.4 °C) is well within the suitable range, but that in Nagano (11.9 °C) is close to the warmer boundary. In Elgin, the Japanese criteria may not be applicable, yet the annual mean temperature (16.3 °C) is high, and the requirement for low temperature, known as chill unit, is not fully met. Chemical treatments are therefore necessary to ensure rest-breaking and achieve satisfactory production in Elgin [11]. Under mild warming of 0.5°C in the future, a threshold level of chill for commercial apple production would be breached in some years even with the chemical rest-breaking [12].

## Methods

This study is based on the field study using semi-structured interviews with full-time apple growers. The semi-structured interviews were carried out with 26 farmers in Nagano from May to June 2009 [17], 40 farmers in Kazuno from June to July 2011 [13], and 11 farmers in Elgin in November 2012 [14].

The farmers were presented with two sets of questions on different aspects of the agricultural practices. The first set of questions concerned the nature of their farming, such as fruit growing areas, major fruit species, cultivars of the fruits, and their typical sales channels. With the second set, they were asked about their experiences of ‘good’ and ‘bad’ years, if any, in their apple farming over the past 30 years, and their perception of any gradual changes or trends noticeable in the production (yields and quality) across the same period. In addition, they were also asked to describe their reactions, if any, to the stimuli they had experienced. The memory of ‘good’ or ‘bad’ years would reflect short-term stimuli such as drop of the sales price or yield loss by climatic hazards, and the noticeable trends would be related to long-term changes including climate change. By making these open-ended questions, rather than asking climate-explicit questions, we aimed to prompt farmers to describe the primary impacts on apple production without a bias toward climate-related events [26]. It was in this context that we tried to understand their adaptations to climatic impacts amidst the exposure to multiple risks.

We also conducted interviews with local experts in apple production to learn about their advices and supports for the farmers on farming practices and sales. In addition, we had discussions with the experts on our interpretations of the farmers’ responses to our questions, such as the relationship between the farmers’ perception of ‘good’ or ‘bad’ years and climatic anomalies, if any. We have thus confirmed that the results of our interviews with farmers have not been exaggerated by extremes, but they would likely represent the responses of the farmers across the individual regions.

## Farmers’ Perceptions of and Adaptation Practices against the Climatic and Non-Climatic Stimuli

Through the interview surveys with farmers, a prominent difference emerged among the farmers regarding their dependence on the farmers’ cooperative with respect to the sales channels and the information sources (Table 2). We therefore categorized the farmers into two groups: a farmer is referred to as a ‘co-op farmer’ when more than a half of the products are sold through the cooperative, otherwise the farmer is a ‘non-co-op farmer’, whose sales channels included direct shipment to individual customers or customer groups via postal or courier service, and direct shipment to retailers and mass market. Table 2 also presents the farmers’ perception of and adaptation to the stimuli, whose details are described below for each study region (see S1–S3 Tables for further details in each region).

**Table 2:** Farmers’ perceptions of and adaptation actions to climatic and non-climatic stimuli subject to the groups followed by the types of adaptation.

		Kazuno		Nagano		Elgin
		Co-op^a^	Non-co-op^a^	Co-op^a^	Non-co-op^a^	Co-op^a^
Perception of incidents and changes	Short-term incidents for good years	-	-	New cultivars	-	Cold winter
				Hazards in other regions		Good currency exchange rates
	Short-term incidents for bad years	Typhoons	Typhoons	Typhoons	Typhoons	Warm winter
		Droughts	Droughts	Hails	Hails	Sunburn
		Hails & frosts	Hails & Frosts			
	Long-term changes with positive effects	-	-	-	-	-
	Long-term changes with negative effects	Fungal disease	Fungal disease	Paler fruits colour	Delayed ripening	Warmer winter
		Decline of apple price	Decline in apple sales	Sunburn	Sunburn	Sunburn
				Pests	Pests	Higher input costs of oil, labour, and fertilizer
Adaptation to climate change	Action taken	Adoption of peach	Introduction of peach	Introduction of better colour strains	Shipment of the *apples without leaf-picking*	Introduction of cultivars requiring less chill unit
				Colour enhancing practices		
	Intent	Autonomous	Autonomous	Planned	Autonomous	Planned
	Timing	Proactive	Proactive	Reactive	Proactive	Reactive
	Direction	Top-down	Bottom-up	Top-down	Bottom-up	Top-down

^a^ A farmer is referred to as a ‘co-op farmer’ when more than a half of their products are sold through the farmers’ cooperative or an equivalent institution, otherwise the farmer is a ‘non-co-op farmer’.

### Kazuno, Japan

#### Farmers’ perceptions of the climatic and non-climatic stimuli in Kazuno

Of the 40 interviewees in Kazuno, 21 were non-co-op farmers and 19 were co-op farmers. Although the two groups were similar in the perception of stimuli, they differed in the reactions. Among the short-term stimuli, all the farmers first recalled typhoons as exerting detrimental damages to the apple production (Table 2). They especially referred to a particular storm that hit northern Japan in September 1991, just before the harvest, which caused a huge yield loss, equivalent to 53% of the total harvest in the previous year [19]. Additional yield losses were also recorded in subsequent years owing to the damages to the trees. In addition to this particular storm, other smaller typhoons were also mentioned.

A drought from July to August in 1994 due to high temperature and low rainfall was recognized by almost all the farmers as a memorable incident that reduced apple production (Table 2). The yield loss occurred in the subsequent year, as the heat and moisture stresses in July led a loss of flower buds in the next year [27]. The yield loss in 1995 amounted to ca. 15% of the total harvest in the previous year [19]. Farmers mentioned that the drought was particularly damaging to high-quality fruits, and subsequently caused even greater loss in income than that in yield.

Hail and frost were also recognized by many farmers (Table 2) as damaging, although the extent of the damages largely depended on the location of the orchard (e.g. the orchards at the foot of mountains were more often exposed to frost). In this region, the earliest frost date is October 22 on the average [21], and the major variety 'Fuji' tends to encounter the frosts because its harvest is from middle to end of November.

For a long term risk the soil-borne fungal disease: violet root rot was mentioned. It has been widely seen ever since the beginning of apple cultivation in this region, and about 67% of apple orchards in Kazuno have been infested at some point [28]. The symptoms include reduced fruit size and yellowing and earlier fall of leaves, which weaken the trees and finally destroy them in a few years. Almost all the farmers have struggled with this disease for a long period, but a failure of the pest control has led to the spread of this disease across entire orchards (Table 2).

Reduced sales were another long term trend that many farmers have observed. This issue was more emphasized by non-co-op farmers than the co-op farmers. The non-co-op farmers stressed the decrease in the size of the sales, especially that of high-quality, i.e. high-price, apples largely reducing their income. The co-op farmers, on the other hand, mentioned the drop of average apple prices at the market leading to their lower income (Table 2).

#### Farmers’ adaptation strategies against the stimuli in Kazuno

In 1992 and 1993, after the typhoon in 1991, the non-co-op farmers started planting peach in a small portion of their orchards as a trial along with other species such as grape, pear and apricot. When the drought in 1994 killed all other species than peach, they confirmed the tolerance of peach against drought, and started enlarging its area to commercial production. Peach is immune to violet root rot, and the harvest is done before the typhoon season. They particularly preferred replacing the early maturity variety of apple ‘Tsugaru’ by peach, since the harvest timing of this apple variety overlapped that for peach, and the price of the apple variety was low anyway. Peach cultivation was first started by four non-co-op farmers, and then during the next five years other 12 non-co-op farmers started peach as well, either because they were recommended by the first four farmers or they observed the success of those who started earlier (Table 2; [13]). About ten years after the peach introduction, the city government and the farmers' cooperative finally started supporting peach production by subsidizing the purchase of the seedlings and establishing the sales channels for peaches, which prompted many farmers, mostly co-op farmers, to adopt peach cultivation (Table 2). As of 2012, ca. 150 out of the 350 apple farmers in this region produced peaches, and the number was still increasing.

Other than starting peach production, purchasing fruit insurance was a common action to mitigate the loss of income by typhoons and hails. Since this region is not regularly hit by typhoons, however, some farmers did not buy the insurance but introduced storm protection nets or took off-farm jobs to compensate for the income loss. They felt that they would pay more for insurance than they would receive in payouts when averaged across years. Against the impacts of drought, on the other hand, no insurance was available; it covers the yield loss caused by the incidents on a single year basis, but not the harvest loss in the following year, which was the case in the drought damage. In the following spring after a drought, when farmers observe far fewer flower buds than usual, they would adopt common farming practices such as very careful fruit thinning in order to maximize the fraction of high quality apples.

Another remedy to the climatic damages, such as typhoons and droughts, for the non-co-op farmers was to process the fruits at their own juice company and to sell them to their customers along with apples. They established the company around 30 years ago to ameliorate the economic losses caused by the shipment of the unsold low-quality fruits to other processing companies at very low prices. Over time, they invented new apple products, e.g. jams, beside juice to promote their sales. They also encouraged other co-op farmers to join this undertaking. For the other farmers without regular customers, however, the lack of a sales channel for the fruit products was the main obstacle, hence only a limited number of farmers joined this company. Other farmers sold apples fell from the trees to other processing companies through the co-op or just discarded them.

Despite the high sensitivity of apples to the violet root rot disease, no preventive measures have been established. Development of the disease can be halted by agronomic practices, e.g. nutrient management, as recent research has indicated [28]. Nevertheless, when an apple tree is severely infested, farmers are forced to uproot it and remove soils in the root zone before planting a new tree, or to keep the affected spot unplanted. If it were not for the pressure from this disease, shifting to peach might not have happened at all or done so more slowly than observed.

### Nagano, Japan

#### Farmers’ perceptions of the climatic and non-climatic stimuli in Nagano

In Nagano, of the 26 interviewees, 13 farmers were co-op farmers and the rest were non-co-op farmers. The main risks observed by the farmers as long term changes included paler color of fruits (co-op farmers), later ripening (non-co-op farmers), and an increase of pests and sunburn of fruits. In Japan, the fruits are highly evaluated with their appearance [29], and for apples bright color is one of the important traits. Paler color fruits get much lower prices, or might be sent to processing. Apples get a red color by being exposed to cold temperature for some duration before harvest [30], and the pale coloring is said to be seen more frequently and widely in Japan owning to increasing temperature [10]. A recent research also reported that changes in taste has been observed in Nagano Prefecture such as an increase in soluble-solids concentration [31]. Such changes in fruit quality may have resulted from earlier blooming [32] and higher temperatures during the maturation period [31]. Non-co-op farmers determined the harvest with the day of first frost in the fall, which, they said, has become later. The sunburn is caused by exposure of fruits to strong sunlight and high temperature in summer; pest infestation may have also been accelerated by high temperature during the growing season as reported for grapes [33]. All these phenomena were mentioned by the farmers in relation to change in climate.

Another event, the typhoon in 1998, was referred as most devastating by almost all the farmers regardless of the groups. It hit this region in October 1998, and dropped from 30 to 60% of fruits on trees just before harvest. Total amount of harvest and sales in that year was anomalously low and this incident was memorized firmly by the farmers [17]. Hail storms have been observed almost every year in this region, but their damage was limited to some farmers or to some portion of the orchards. Only the farmers who have often experienced hail counted it among serious disasters [17].

As incidents that made a ‘good’ year, co-op farmers mentioned introduction of new cultivars into market and thereby increase in the sales. The most recent such incident was around the year 2000, when the new cultivars: *Shinano Sweet* and *Shinano Gold* received high prices and the farmers’ income rose for the subsequent three to four years. Hazards in other production areas were also mentioned by co-op farmers in relation to a ‘good’ year. In 2007, for example, Aomori prefecture: the other big producer of apples in Japan, was hit by a typhoon, and the market price rose by 5% above the previous year [19]. This event brought higher income to the farmers in Nagano and was remembered as being ‘good’. Interestingly, mention of these events, i.e. new cultivars and hazards in other regions, was not seen among the non-co-op farmers in relation to ‘good’ years (Table 2; [17]).

#### Farmers’ adaptation strategies against the stimuli in Nagano

In order to accelerate the coloring of apple, co-op farmers followed the experts’ advises to better expose the fruits to full sunlight by picking off the leaves around the fruits, turning around the fruits, and laying reflective materials on the orchard’s ground (Table 2). Additionally, some farmers practiced bagging: the fruits are covered by a paper bag from shortly after fruition until one month before harvest. This practice was done originally to protect apples from insects [34], but is done now mainly to get good appearance with bright red color. Another action taken against paler coloring was to select genotypes with better coloring. With apples many new strains have been identified among natural mutations of original cultivars [35], and in Japan some of the new strains with better coloring have become available for the farmers. Co-op farmers tended to adopt the new strains and cultivars with better coloring along with the color accelerating practices (Table 2).

Non-co-op farmers, on the other hand, opted for keeping the cultivars close to the original one over changing to the new ones, since their customers preferred them for their better taste to the new ones. They did not practice the color-enhancement measures either. They mentioned that picking the leaves around fruits would hinder sugar accumulation in fruits, and that the reflective mulching would promote coloring without concomitant ripening, resulting in red unripe fruits. A research indeed showed that foil film enhanced the color of apple without any effects on other quality traits [36]. Without leaf-picking, the fruits could bear shadows of the leaves in paler color on the surface. The farmers then started selling their fruits as *apples without leaf-picking*, which signifies higher sugar content despite the remaining patches of paler color. In response to the later ripening, they simply delayed the harvest through to full maturity. They said that harvest date has become later by about two weeks for the last 20 years. Since the fruits were harvested later, delayed coloring was not recognized as a major change (Table 2). Omission of the leaf-picking also saved on labor for the farmers.

Against typhoons and hails, both groups of farmers bought fruit insurance to mitigate the economic loss, while only a few farmers introduced storm protection nets. In this region, individual farmer has orchards scattered at multiple locations, and introducing the protection net in all the fields would cost more than the benefit. They thus preferred purchasing the crop insurance without installing the protection measure.

### Elgin, South Africa

#### Farmers’ perceptions of the climatic and non-climatic stimuli in Elgin

In Elgin, the farmers depend on three companies for their sales, who collect, pack and ship the fruits to the domestic and international markets. These companies started as farmers’ cooperatives around 1950, and became the private enterprises around 1990. They still retain the basic features of farmers’ cooperatives: farmers kept supplying apples to the same company as they did before and the number of farms supplying to the companies has little changed. All 11 farmers we interviewed supplied their products to one of the three companies [14]. We therefore categorized them as co-op farmers. Almost all the farmers in this region are said to be co-op farmers (personal communication).

The farmers’ perceptions of the stimuli were closely related to temperature in austral winter (Table 2; [14]). In warmer winter, with insufficient chill units, dormancy is broken in a smaller number of buds, and the trees bear less fruit to yield poor harvest. Furthermore the profit was closely linked to the number of harvested apples with the fruit quality making little differences. The poor production years thus brought lower income. The years 2010 and 2011 were, for example, referred to as bad production years, hence low profit, due to the warmer austral winters in 2009 and 2010 respectively. On the other hand, colder winters with higher chill units brought good harvest and good profit, which was observed recently in the harvest of 2008, 2009 and 2012. For the long-term, farmers felt that winter is getting warmer resulting in even fewer chill units. The increase in the cost of inputs such as oil, labor, and fertilizer, and shipping cost were also referred to as tightening their profit margins (Table 2).

One specific year, 2008, was mentioned as being ‘good’ by all the farmers and experts. From 2007 to 2008, the South African Rand dropped dramatically in value against Euro and Pound Sterling: the currency exchange rate was R 9.5 to 1 Euro in 2007, but became R 13–14 to 1 Euro in December 2008 [37]. This change of currency exchange rate, accompanied by the good harvest as noted above, made 2008 a memorably good year for the farmers in this region (Table 2), where more than 40% of apples are exported (section 2).

Other stimuli which led to poor production year included heat waves in austral summer, such as the one in January 2011, and hail damage. Heat waves caused sunburn in the fruits and gave water stress to trees resulting in smaller size of the fruit. Sunburned fruits were sent to processing and juice making, where fruit received much lower prices resulting in farmers’ loss of income. The damage by hail largely depends on the location of the farm and orchard, but generally hail after flowering reduced the yield most.

#### Farmers’ adaptation strategies against stimuli in Elgin

Against the insufficient chill units, farmers were advised to change the recipes of the chemicals for breaking dormancy, such as the mix ratio of oil and Cyanamid, depending on the record of chill units [14]. Breeding for new cultivars with lower chill requirement has been undertaken, and some of them might be widely accepted by the farmers. The current major cultivar, Granny Smith, requires fewer chill units, but is prone to sunburn. It is recommended to protect this cultivar from sunburn by installing shade nets, the cost of which, however, exceeded the profit gained, according to the farmers. They rather introduced cultivars other than Granny Smith when replanting their orchards and the shift in cultivars has already been seen [14]. Popular cultivars were Cripps’ Pink and Fuji; also because they get high price at the markets. Indeed, young trees of Granny Smith (0 to 10 years) contribute much less (10% of total Granny Smith) than old trees (more than 25 years, 67% of total) and the area for Cripps’ Pink has increased by about 30% in the last three years [20]. Many farmers plant other fruits such as pears, where the chill unit requirement is generally less than that of apples. However, apples generate higher income than other fruits and farmers do not plan to decrease the area of apples at present.

## Characterization of the Adapting Actions

The three cases illustrated diverse actions against the stimuli including climate change and variability. The various adaptations can be characterized along three axes, viz.

Intent: autonomous or planned in their purposefulness,

Timing: reactive, concurrent or proactive in relation to occurrence of the change [16], and

Direction: top-down or bottom-up at the origin of the action.

The adaptation undertaken by the non-co-op farmers in Kazuno, i.e. introduction of peach, was bottom-up, autonomous and reactive to both climatic hazards (short-term stimuli) and non-climatic stressors, e.g. fungal disease. It could also turn out to be a proactive yet unintentional or incidental adaptation to long-term climate change (Table 2) [16]. Also, the sales of *apples without leaf-picking* started by the non-co-op farmers in Nagano were bottom-up, autonomous and proactive to the effects of climate change, particularly to the paler color (Table 2). In Elgin, on the other hand, the action was top-down, concurrent and reactive, and planned to climate change especially to rising temperature (Table 2). Farmers and experts were aware of the climatic trend and its effects, and responded to the stimuli. Situation was similar in Nagano among the co-op farmers, whose actions were concurrent and reactive, and planned to offset the effects of climate change.

In our cases, therefore, non-co-op farmers initiated bottom-up actions which serve as proactive adaptation to climate change, whereas co-op farmers followed institution led top-down actions which were reactive. The co-op farmers’ adoption of peach cultivation in Kazuno was proactive to climate change, but they only followed the adaptation initiated by the non-co-op farmers. It thus appears that the type of adaptation depends on those who initiated the actions. We therefore focus on the social actors: individual farmers or institutions, who initiated the adaptation actions, and investigate their roles in the adaptation process, which is yet to be studied or understood [38]. We further explore the possibilities of linking the actions of institutions and individuals.

### Farmer led bottom-up adaptations

The bottom-up adaptations: introduction of peach production in Kazuno and sales of the *apples without leaf-picking* in Nagano, were both undertaken by the farmers of non-co-op groups, and are characterized by the following three features in common.

First, the non-co-op farmers in either case had their own channels for shipment of the products to the customers, such as individual consumers, consumer groups, and retailers. Through these channels, they also got responses of the customers on their products. Facing the customers’ evaluation and sometimes critiques, they would have been motivated to make changes for better satisfaction of the customers and thereby to secure the continued purchase. To this end, the farmers explored new products seeking the customers’ preferences by trial-and-errors [38].

The *apples without leaf-picking* in Nagano was a trial to gain the customers’ acceptance of the color anomaly on the fruits. As mentioned earlier, the color enhancement practices are often at odds with the farmers’ efforts to provide tasty fruits to customers. After getting complaints from the customers that the fruits were not tasty despite good appearance with bright color, the farmers started omitting the color enhancement, waiting for full ripeness and shipping the fruits under the new name of *apples without leaf-picking*. Such fruits would have been priced very low despite the good taste when shipped to the mass market. Having the direct link with the customers, the farmers were able to gain the acceptance of the *apples without leaf-picking*. This trial has eventually led non-co-op farmers to secure the niche market and made them less prone to the color deterioration while omitting the labor-intensive practices of leaf-picking.

The peach introduction in Kazuno was a trial to confirm the customers’ preference. The small number of non-co-op farmers grew various other species than apple and sent the fruits to their regular customers for trials, which indicated peaches as being most favored by the customers. This encouraged the small number of non-co-op farmers to grow peaches on a production basis, which spread among other apple growers to eventually establish the niche at the mass market [13]. The process of this bottom-up adaptation to climate change is comparable to the process of decentralized diffusion of innovations [13], where a limited number of people initiate something new, which are then adopted by increasing number of people [39].

Similar cases that the direct connection with the customers served as both challenges and chances for the farmers have also been reported elsewhere. In Austria, family farmers in particular are engaged in direct-marketing and have built direct contacts with other stakeholders to get information and networks [40]. In the southeast of England, farmers captured niche market by testing out new crops: for example walnuts are now grown by a stone fruit farmer [41]. The direct connection with individual customers would hence led farmers to capture the niche market.

Second, the non-co-op farmers had significantly larger areas for cultivation than the co-op farmers in Japan [13, 17]. The former group of farmers can therefore better afford to investigate in and take the risks of the new trials, of which most would end up in vain. It is reported that adaptations undertaken by coffee farmers in Latin America are more likely related to the availability of land rather than their perception of risks posed by climatic and non-climatic stimuli [42]. In their study, farmers also perceived the price volatility of coffee and weather fluctuations as being inherent to their farming [42]. Farmers with larger land area may have more spare land to prepare for the fluctuations and changes, and would more likely to make the changes in their farming practices as per their recognition of the necessity. The better endowment of the resources needed to take the risk of starting something new is recognized as a common feature of the *innovators* [39].

Third, bottom-up adaptive actions were initiated by a small number of individual farmers. In Kazuno, the peach was first introduced by four non-co-op farmers. In Nagano, only a few non-co-op farmers started the *apples without leaf-picking*. In either case, the small number of farmers visited each other’s orchards very frequently to exchange information about farming or sales before starting the adaptive actions. The situation may have been similar to that for wine grape growers in northern California, where most adaptive decisions were made individually, and a collective action has been undertaken only to respond to a large-scale pest outbreak [43].

### Institution led top-down adaptations

In Elgin and Nagano, the adaptation by color-enhancing practices and adoption of new strains were introduced by the institutions, i.e. national and local research institutes and accepted by the farmers. These actions are arguably progressive rather than innovative as compared with the farmer led adaptation in 5.1. We argue that the institution led adaptations are determined by the farmers’ needs, the established sales channels, and the process of priority setting within the institutions.

Where negative climatic impacts are pervasive, like in Elgin and Nagano, the institutional adaptation policy would focus on the most salient issue whose negative impacts must be reduced as soon as possible. This would lead to reactive or concurrent actions. Most farmers would also opt for accepting the institutional initiative rather than taking risks of trying something new by themselves. The decision on the adaptation measures would hence be made under time constraint that would restrict the options for trying out. As seen in Elgin and Nagano, the institutions put the priority on finding new cultivars with lower chill requirement (Elgin) and on introducing the farming practices for enhanced coloring (Nagano), which also required only moderate changes to the current practices. Hence these options matched the farmers’ needs and were accepted quickly. Their wide acceptance among the farmers across the region conforms well to the diffusion of *centralized* innovation [39], which takes place when the priority of policy makers matches that of farmers.

In addition to the institutional policy priority, stability of the sales channels may dictate the institutional actions for adaptation. For the co-ops, securing the sales channels for a large-scale shipment of fruits is at the high priority, and they would not risk changing the channels unless they are strongly forced to. In Elgin, about a half of the co-op’s sales is exported to Europe and other African countries. This sales channel is relatively secure, since the apple harvest time in South Africa does not overlap with those in the northern hemisphere. South Africa is the fourth largest apple producer in the southern hemisphere after Brazil, Chile and Argentina [18], and the co-ops have so far secured the export markets by supplying the apples with the required quality, e.g. size and color. With this secure sales channel, both the co-ops and the co-op farmers would strive to produce apples by adopting agronomic practices against the marginal climate. Elgin is also a major production site of pears and grapes [20], but the farmers and the co-ops we interviewed intended to maintain apples as the major products, since the price for apples are better than the other fruits. A similar situation can be found in Nagano, where the climatic margin is approaching but, being the second largest apple producer in Japan, they try to keep securing the domestic market with introducing the agronomic practices against the deteriorating color.

At the last, the institution led adaptation is inherently limited for its ranges of scope. For the institutions in Kazuno, starting peach production could not have been an option when the four farmers started it around 1992. This region has not been subjected to the negative effects of climate change except for individual climatic hazards. Rather, the climate was quite suitable for apple production, but was considered to be too cool for quality peach production. Indeed the institutions only started supporting peach production after certain number of farmers had explored the niche at the market. In Nagano, the fruit with shadow of leaves could never be an option for institutional action, since they would be poorly evaluated at the mass market where the evaluation with appearances dominates [29]. Without a sizeable market for such fruits, the idea was not considered or supported by the institution in reason.

### Optimizing adaptations by combining the bottom-up and top-down approaches

As discussed above, the bottom-up and top-down adaptations have contrasting features. Nevertheless, they are not mutually exclusive in nature, and could complement each other. In Kazuno, indeed, the both types of adaptations happened contingently, resulting in the capture of niche market. This is, as an innovation process, a combination of bottom-up beginning followed by top-down institutionally-supported diffusion [39].

In the institution led adaptation, as has been discussed in the robust adaptation decision making [44], options should be screened and appraised to eventually choose the best one among them [44]. This framework might be applicable to or even the only choice for adaptation with large-scale engineering work, but it is not practical for the small-scale actions like in our cases. These actions at the initial stages are too small to be considered as an option in the decision making process, but some of them may later turn out to be successful innovations that cannot be attained by top-down adaptations. It must therefore be recognized that adaptation to climate change should not be confined to institution led top-down actions, but that it should also include the individually initiated bottom-up actions. Since the two types of actions have their own limitations and assets, they should ideally be combined, as in Kazuno, for better chances of successful adaptations. To this end, roles of the various actors, particularly those of a small number of non-co-op farmers must be better recognized.

It must also be pointed out that not all the observed adaptations are deliberately planned as adaptive actions against climate change, but that some are by-products or secondary benefits from activities unrelated to climate change [41]. In many cases climate change may have been used as a means of justifying a change that would otherwise be seen as less benign (such as cost-cutting) [41]. Also, success in the near term may turn out to be maladaptive in the long run, and vice versa [45]. In Nagano as well, the *apples without leaf-picking* was not intended to mitigate the challenge of paler color but to improve the quality of fruits and to omit extra labor. Later on, nevertheless, it turned out to be a viable option for adaptation. It is therefore advised not to confine the adaptations to the countermeasures against climate change impacts, but to recognize them as a part of the farmers’ and the institutions’ efforts against multiple and interacting challenges.

## Implications

Focusing on the social actors: individual farmers and institutions, we argue that a combination of the farmers-initiated bottom-up and the institution-led top-down approaches would facilitate more flexible and widely-accepted adaptations to climate change. The involvement of a diversity of actors could make the entire adaptation more dynamic and innovative. Diversity of the actors would notably vary much by regions and societies, and so does the scope of the adaptations. Recent adaptation studies, being urged by the greater necessity to develop and implement adaptation policies, have put a high priority on assessing vulnerability and defining vulnerability indicators [46]. The findings of this study are, however, at odds with the idea of capturing the vulnerability with a set of versatile indicators for policy making. Adaptation is a dynamic process driven by multiple actors, which depends very much on the society of concern. It would be more fruitful to promote the proper combination of top-down and bottom-up adaptations which might lead more *resilient* [47] adaptations.

Finally, we emphasize that more field based cases must be studied for comprehensive understandings of the adaptation processes. We hope that, with an accumulation of case studies on various agricultural crops and systems across the regions, adaptation science would be able to provide substantial feedbacks to adaptation policy in agriculture.

## Supporting Information

S1 Table Results of the interview survey with farmers in Kazuno. *C signifies co-op farmers and N signifies non-co-op farmers.
(DOCX)

S2 Table Results of the interview survey with farmers in Nagano. *C signifies co-op farmers and N signifies non-co-op farmers.
(DOCX)

S3 Table Results of the interview survey with farmers in Elgin. *C signifies co-op farmers and N signifies non-co-op farmers.
(DOCX)
