# Supplementary material for: What Drives Farmers to Make Top-Down or Bottom-Up Adaptation to Climate Change and Fluctuations? A Comparative Study on 3 Cases of Apple Farming in Japan and South Africa
Source: PLoS One. 2015 Mar 30;10(3):e0120563. doi: 10.1371/journal.pone.0120563 (PMC4378992; doi:10.1371/journal.pone.0120563)
Supplement: S2 Table — *C signifies co-op farmers and N signifies non-co-op farmers. (DOCX) [file pone.0120563.s002.docx]

S2 Table. Results of the interview survey with farmers in Nagano. *C signifies co-op farmers and N signifies non-co-op farmers.

| No | Group* | Area planted | | Perception of incidents and changes | | | | | | | | Adaptation actions | | |
| --- | --- | --- | --- | --- | --- | --- | --- | --- | --- | --- | --- | --- | --- | --- |
|  |  | >1.5ha | <1.5ha | Hazards in other regions | New Cultivars | Typh-oons | Hails | Sunburn | Pests | Delayed ripening | Paler color | Better color strains | Color enhancing practices | Apples without leaf-picking |
| 1 | C | ✓ |  |  | ✓ | ✓ |  | ✓ | ✓ |  |  | ✓ | ✓ |  |
| 2 | C | ✓ |  |  |  | ✓ | ✓ | ✓ | ✓ |  |  | ✓ | ✓ |  |
| 3 | C | ✓ |  | ✓ | ✓ | ✓ | ✓ |  | ✓ |  | ✓ | ✓ | ✓ |  |
| 4 | C | ✓ |  |  |  | ✓ |  |  |  |  | ✓ | ✓ | ✓ |  |
| 5 | C | ✓ |  |  | ✓ | ✓ |  |  |  | ✓ | ✓ | ✓ | ✓ |  |
| 6 | C |  | ✓ | ✓ |  | ✓ |  | ✓ |  |  | ✓ | ✓ | ✓ |  |
| 7 | C |  | ✓ |  |  | ✓ |  |  | ✓ |  |  | ✓ | ✓ |  |
| 8 | C |  | ✓ | ✓ |  | ✓ |  | ✓ |  |  | ✓ | ✓ | ✓ |  |
| 9 | C |  | ✓ |  |  | ✓ |  |  | ✓ |  | ✓ |  | ✓ |  |
| 10 | C |  | ✓ |  |  |  | ✓ |  |  |  |  |  | ✓ |  |
| 11 | C |  | ✓ |  | ✓ | ✓ |  |  |  |  | ✓ | ✓ | ✓ |  |
| 12 | C |  | ✓ | ✓ |  | ✓ |  |  |  |  | ✓ | ✓ | ✓ |  |
| 13 | C |  | ✓ | ✓ |  | ✓ |  |  |  |  |  | ✓ | ✓ |  |
| 14 | N | ✓ |  | ✓ |  | ✓ |  |  |  |  |  |  |  | ✓ |
| 15 | N | ✓ |  |  | ✓ | ✓ |  | ✓ | ✓ |  |  |  |  | ✓ |
| 16 | N | ✓ |  |  |  | ✓ |  |  |  |  |  |  |  |  |
| 17 | N | ✓ |  |  |  | ✓ |  |  |  |  |  |  |  | ✓ |
| 18 | N | ✓ |  |  |  | ✓ | ✓ |  |  |  | ✓ |  |  | ✓ |
| 19 | N | ✓ |  |  |  | ✓ | ✓ |  |  |  |  |  |  | ✓ |
| 20 | N | ✓ |  |  |  |  | ✓ |  |  | ✓ |  |  |  | ✓ |
| 21 | N | ✓ |  |  |  | ✓ |  |  |  | ✓ |  |  |  | ✓ |
| 22 | N | ✓ |  |  |  | ✓ |  |  |  | ✓ |  |  | ✓ |  |
| 23 | N | ✓ |  |  |  | ✓ |  |  |  | ✓ | ✓ |  |  | ✓ |
| 24 | N | ✓ |  |  |  | ✓ | ✓ | ✓ | ✓ | ✓ |  |  |  | ✓ |
| 25 | N | ✓ |  |  |  | ✓ | ✓ |  | ✓ |  |  |  |  | ✓ |
| 26 | N |  | ✓ |  |  | ✓ |  | ✓ |  | ✓ |  |  |  | ✓ |
