# Supplementary material for: What Drives Farmers to Make Top-Down or Bottom-Up Adaptation to Climate Change and Fluctuations? A Comparative Study on 3 Cases of Apple Farming in Japan and South Africa
Source: PLoS One. 2015 Mar 30;10(3):e0120563. doi: 10.1371/journal.pone.0120563 (PMC4378992; doi:10.1371/journal.pone.0120563)
Supplement: S3 Table — *C signifies co-op farmers and N signifies non-co-op farmers. (DOCX) [file pone.0120563.s003.docx]

S3 Table. Results of the interview survey with farmers in Elgin. *C signifies co-op farmers and N signifies non-co-op farmers.

| No | Group* | Area planted | | Perception of incidents and changes | | | | | Adaptation actions |
| --- | --- | --- | --- | --- | --- | --- | --- | --- | --- |
|  |  |  |  | Positive incidents | | Negative incidents | | |  |
|  |  | >100ha | <100ha | Cold winter | Good currency exchange rate | Warm winter | Sunburn | Higher input costs | Change cultivars |
| 1 | C | ✓ |  | ✓ | ✓ | ✓ | ✓ | ✓ |  |
| 2 | C | ✓ |  | ✓ | ✓ | ✓ | ✓ | ✓ |  |
| 3 | C | ✓ |  | ✓ | ✓ | ✓ | ✓ | ✓ | ✓ |
| 4 | C | ✓ |  | ✓ | ✓ | ✓ | ✓ | ✓ | ✓ |
| 5 | C | ✓ |  | ✓ | ✓ | ✓ |  | ✓ |  |
| 6 | C | ✓ |  | ✓ | ✓ | ✓ | ✓ | ✓ | ✓ |
| 7 | C | ✓ |  | ✓ | ✓ | ✓ | ✓ | ✓ | ✓ |
| 8 | C | ✓ |  | ✓ | ✓ | ✓ | ✓ | ✓ | ✓ |
| 9 | C |  | ✓ | ✓ | ✓ | ✓ | ✓ |  | ✓ |
| 10 | C |  | ✓ | ✓ | ✓ | ✓ | ✓ | ✓ | ✓ |
| 11 | C |  | ✓ | ✓ | ✓ | ✓ | ✓ |  | ✓ |
